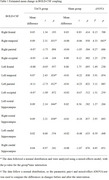# Impact of 40Hz tACS on regional glymphatic flow in patients with mild Alzheimer's disease

**DOI:** 10.1002/alz70856_105318

**Published:** 2026-01-08

**Authors:** Yuanyuan Lu, Yi Xing, Yi Tang

**Affiliations:** ^1^ Innovation Center for Neurological Disorders, Department of Neurology, Xuan Wu Hospital, Capital Medical University, Beijing, Beijing, China; ^2^ Department of Neurology & Innovation Center for Neurological Disorders, Xuanwu Hospital, Capital Medical University, National Center for Neurological Disorders, Beijing, China, Beijing, Beijing, China; ^3^ Department of Neurology & Innovation Center for Neurological Disorders, Xuanwu Hospital, Capital Medical University, National Center for Neurological Disorders, Beijing, Beijing, China

## Abstract

**Background:**

Gamma transcranial alternating current stimulation (tACS) has shown promise in enhancing cognitive function in patients with Alzheimer's disease (AD). However, the impact of gamma tACS on regional glymphatic flow remain unclear.

**Method:**

A total of 46 patients with mild AD were randomly assigned in a 1:1 ratio to receive either 30 one‐hour sessions of 40Hz (gamma) tACS or sham stimulation over 15 consecutive days (Clinical Trial: NCT03920826). Global blood oxygen level‐dependent (BOLD) signals and cerebrospinal fluid (CSF) inflow coupling were measured using resting‐state functional MRI to evaluate glymphatic flow, which was acquired at baseline and right after the intervention, along with cognitive assessments.

**Results:**

Compared to baseline, patients in the tACS group exhibited increased BOLD‐CSF coupling in the left frontal lobe, left posterior lobe, right posterior lobe, and bilateral rostral hippocampus at the end of the intervention. Additionally, changes in regional glymphatic flow in the right temporal lobe, right parietal lobe, and bilateral rostral hippocampus showed a positive correlation with changes in cognitive assessments.

**Conclusion:**

The findings demonstrated the beneficial effects of gamma tACS on regional brain glymphatic flow, which might represent a potential therapeutic mechanism of tACS.